# Violence Experienced by Nursing Students During Clinical Practice and Academic and Emotional Consequences: A Cross-Sectional Study

**DOI:** 10.3390/nursrep16050167

**Published:** 2026-05-15

**Authors:** Samantha Ruth Novales-Huidobro, Maria Lorena Ángeles-Pacheco, Misato González-Kawahara, Natalia Constantino-Segura, Paula García-Olea, Reyna Sámano, Gabriela Chico-Barba

**Affiliations:** 1 School of Nursing, Universidad Panamericana, Augusto Rodín 498, Insurgentes Mixcoac, Benito Juárez, Ciudad de México 03920, Mexico; snovales@up.edu.mx (S.R.N.-H.);; 2 Coordinación de Nutrición y Bioprogramación, Instituto Nacional de Perinatología, Montes Urales 800, Lomas de Virreyes, Miguel Hidalgo, Ciudad de México 11000, Mexico

**Keywords:** clinical training, clinical learning environment, workplace violence, nursing students, professional development

## Abstract

**Background**: Violence in healthcare settings affects nursing students during clinical training and may compromise their mental well-being, learning experiences, and professional development. Despite evidence from high-income countries, limited data exist on how contextual and organizational factors in Latin American settings shape these experiences. This study aimed to assess the frequency and types of violence experienced by nursing students during clinical practice and the academic and emotional consequences. **Methods**: Cross-sectional study conducted among undergraduate and graduate nursing students in Mexico City who had completed at least one hospital-based clinical placement in the previous 12 months. Data were collected between January 2024 and September 2025 using a validated questionnaire assessing types of violence, perpetrators, academic and emotional consequences. Violence was defined as experiencing events “occasionally,” “sometimes,” or “frequently.” Descriptive statistics were calculated. Associations were examined using Pearson’s chi-square test, and logistic regression models adjusted for age, sex, and year of study. **Results**: Seventy-three students participated (86.3% female). Non-physical violence was the most frequent type (90.4%), followed by sexual harassment (49.3%), mainly perpetrated by nurses (62%) and physicians (46.5%). Considering leaving the profession (41.4%) and feelings of inadequacy (66.2%) were the most common academic and emotional consequences. Although some associations were observed in bivariate analyses, these were not significant after adjustment. **Conclusions**: Violence during clinical training is highly prevalent and may represent a significant threat to nursing students’ mental well-being and professional development. Strengthening institutional policies, reporting mechanisms, and supportive learning environments is essential to mitigate its impact and promote safer clinical training.

## 1. Introduction

Violence in healthcare and educational settings is increasingly recognized as a critical issue, with significant implications for nursing education and professional development [1]. According to the World Health Organization (WHO), between 8% and 38% of health professionals report experiencing physical violence during their careers [2], while another study indicates that around 58.7% have been exposed to some form of aggression in the clinical environment, most commonly verbal or psychological [3]. Among nursing students, non-physical forms of violence—such as verbal abuse, intimidation, and psychological aggression—are consistently reported as the most prevalent, with documented consequences including emotional distress, decreased self-confidence, and intentions to leave the profession. Nursing students, in particular, are among the most vulnerable groups during clinical practice; their limited clinical experience, dependence on evaluation by supervisors, and lower position within institutional hierarchies may reduce their ability to recognize, report, or respond to violent situations [1,4,5]. Rather than supporting experiential learning, some clinical settings expose students to repeated verbal hostility, intimidation, or sexual harassment, which threaten both their psychological safety and their capacity to learn effectively [1,6,7].

Workplace violence in clinical training environments cannot be fully understood without considering the organizational and hierarchical structures in which it occurs. Healthcare and educational settings are typically characterized by marked power asymmetries, where students occupy subordinate positions within rigid hierarchies, potentially limiting their autonomy and capacity to respond to adverse situations [5,8]. These power dynamics may influence not only learning experiences but also the ways in which violence is perceived, tolerated, or normalized [9].

Previous research has shown that hierarchical organizational models can restrict decision-making and autonomy among nursing professionals [8], while power imbalances in nursing education may lead students to silence, compliance, or reduced participation [5]. In parallel, workplace violence has been described as a frequent phenomenon in clinical training contexts, with evidence suggesting that it may become normalized as part of professional socialization processes [9].

Within this framework, recent evidence indicates that exposure to violence during clinical practice adversely affects nursing students’ professional development and well-being [1,6]. This exposure is particularly detrimental during clinical placements, where observation, mentorship, and feedback are essential components of professional socialization [10]. Behaviors observed and experienced in clinical settings may be internalized by students and become part of the hidden curriculum, indirectly shaping their perceptions of professional norms, roles and interpersonal conduct beyond the formal curriculum [11]. Such normalization, reinforced through hierarchical and institutional dynamics, may perpetuate intergenerational patterns of mistreatment within the nursing profession [9,12].

Despite an expanding body of literature documenting the prevalence of violence among nursing students in North America (e.g., the United States and Canada), Europe (e.g., United Kingdom, Italy, Finland), Asia (e.g., Republic of Korea, China, Iran, Israel), Australia and Africa (e.g., Ghana and South Africa) [1,6,7,13], important gaps remain in understanding how this phenomenon is shaped by context. Most available evidence originates from high-income countries, and data from Latin American settings remain limited [7]. This underrepresentation is important because violence during clinical training is not only an interpersonal problem; it is also shaped by organizational hierarchies, professional role expectations, workplace culture, and reporting practices. In Latin American settings, these factors may influence both the manifestation of violence and how such experiences are interpreted and reported by students [1,4].

Although numerous systematic reviews have been conducted [6,7,13], few Latin American studies have been included, likely due to language restrictions and publication bias, resulting in a predominance of evidence from high-income countries. Furthermore, differences in nurses’ scope of practice and levels of professional autonomy between high-income countries—such as the United States or the United Kingdom—and Latin American contexts [14] may influence the frequency, forms, and reporting patterns of violence experienced by nursing students during clinical practice. In Mexico specifically, where nursing education often takes place in highly hierarchical clinical environments and nursing roles continue to evolve in terms of autonomy and professional recognition, these structural characteristics may shape how students experience, interpret, and report violence. The limited availability of region-specific evidence makes it difficult to determine whether patterns observed in high-income countries are directly applicable to Latin American contexts.

The predominance of evidence from high-income settings limits the capacity to design targeted educational interventions that promote safe and supportive clinical learning environments culturally and institutionally relevant to Latin American contexts. Examining violence in Mexico is therefore relevant not only to address a regional evidence gap, but also to strengthen the international knowledge base on how context shapes unsafe clinical training environments. Therefore, this study aimed to identify the frequency and types of violence experienced by nursing students during their clinical practice in Mexico and the academic and emotional consequences. Based on previous studies reporting high prevalence rates of violence during clinical training and identifying non-physical violence as the most common form, we hypothesized that nursing students would report a high frequency of violence during clinical practice, particularly non-physical violence.

## 2. Materials and Methods

### 2.1. Study Design and Setting

A cross-sectional study was conducted at the School of Nursing, Universidad Panamericana, and the Instituto Nacional de Perinatología in Mexico City from January 2024 to September 2025.

### 2.2. Sample and Recruitment

Participants were recruited through convenience sampling. This approach was chosen to include the entire accessible population of nursing students who met the inclusion criteria within the participating institutions, which have a defined student population, allowing comprehensive coverage of the target group.

Participants met the following inclusion criteria: undergraduate and graduate nursing students, as well as nursing interns, who had completed at least one hospital-based clinical practice within the previous 12 months to reduce recall bias. All eligible students were invited to participate in the study, with a participation rate of 92.4%.

No formal sample size calculation was performed, as the study aimed to include the total accessible population during the recruitment period. Therefore, the final sample reflects all eligible participants available within the study context. This approach prioritizes coverage of the target population; however, it may limit the statistical power to detect associations in inferential analyses.

### 2.3. Data Collection and Instruments

An electronic questionnaire was administered in group sessions, during which participants were informed about the objectives of the study. Two research team members were present solely to provide clarification on questionnaire items and to assist with any technical issues during completion. Participants completed the questionnaire anonymously via SurveyMonkey using their own devices. Researchers did not have access to participants’ responses and did not observe their screens during completion. No personally identifiable information was collected, and responses were not accessible to instructors or academic evaluators. Participants were informed that their responses would remain confidential and would not influence their academic evaluation, in order to encourage honest reporting.

Sociodemographic and academic variables, such as age, gender, and year of study, were obtained through direct questions. The instrument used to identify the frequency and types of violence experienced by nursing students in their clinical practice during the past year was originally developed by Hewett [15] and subsequently adapted and validated for the Mexican population, demonstrating internal consistency (Cronbach’s α = 0.89) [4].

The instrument consisted of 66 items divided into five sections: (1) sociodemographic data; (2) experiences of violence, including non-physical violence, physical violence and sexual harassment; (3) perpetrators of violence, impact on academic performance, and emotional consequences; (4) reporting of violent incidents; and (5) management of violence in the clinical setting. These domains were defined in the original instrument and retained in the adapted version, as they allow for a more comprehensive assessment of both the occurrence of violence and its impact on students.

**Figure 1:**
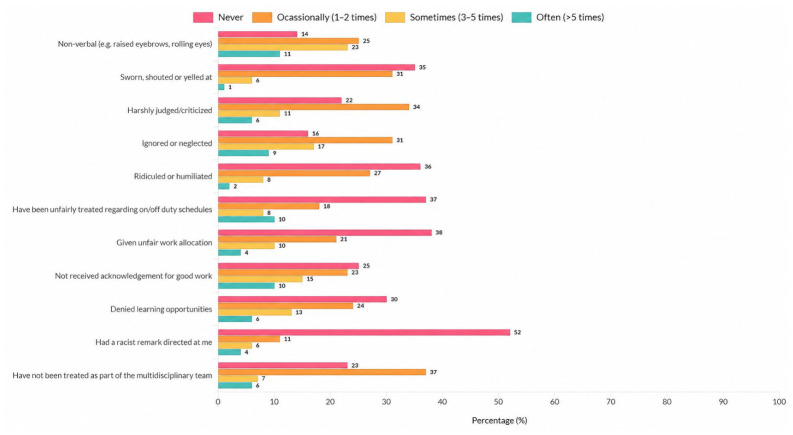
Distribution of non-physical violence types experienced by nursing students during clinical practice (*n* = 73).

**Figure 2:**
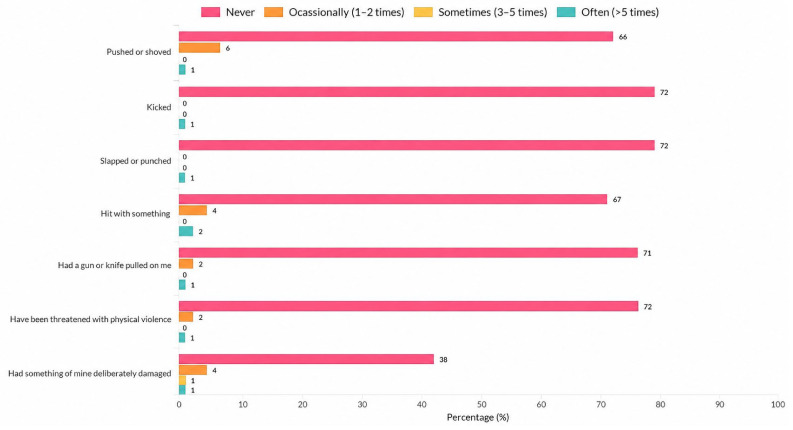
Distribution of physical violence types experienced by nursing students during clinical practice (*n* = 73).

**Figure 3:**
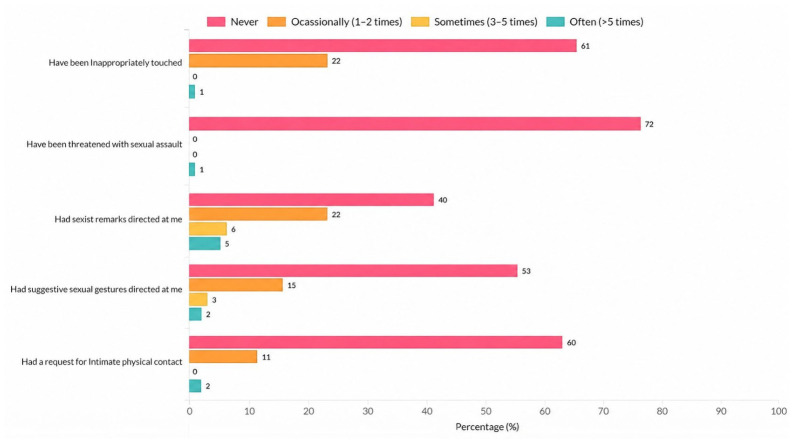
Distribution of sexual harassment types experienced by nursing students during clinical practice (*n* = 73).

**Figure 4:**
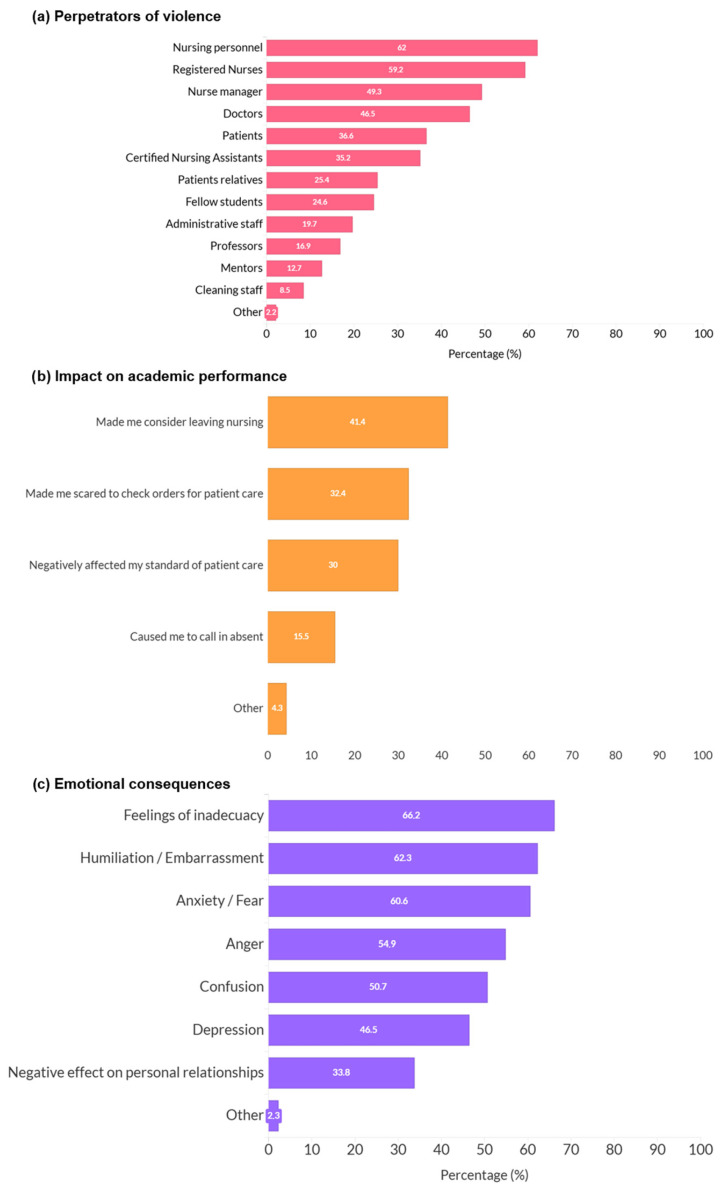
Perpetrators and consequences of violence during clinical practice among nursing students (*n* = 73). Note: The number of responses varied slightly across items due to missing data (*n* = 71–73).

Section 2 included items assessing specific violent experiences during clinical practice, such as being ignored or excluded, being shouted at, being pushed or hit, and being exposed to sexist remarks or unwanted sexual gestures. Section 3 assessed who perpetrated the violence (e.g., nurses, physicians, patients, or relatives), as well as its academic and emotional consequences, including considering leaving nursing, feeling scared to check orders for patient care, feelings of inadequacy, humiliation, embarrassment, anxiety, or fear. The full set of items assessing types of violence, perpetrators, and consequences are presented in detail in Figure 1, Figure 2, Figure 3 and Figure 4.

Section 2 and Section 3 included Likert-type questions that assessed frequency using a four-point scale: never (0 times), occasionally (1–2 times), sometimes (3–5 times), and frequently (>5 times). Section 4 included dichotomous questions assessing agreement (agree/disagree). Section 5 consisted of an open-ended question requesting recommendations for improving the management of violence in clinical training environments. All responses were exported from SurveyMonkey into a SPSS file for statistical analysis.

### 2.4. Statistical Analyses

Sample characteristics were described using median and interquartile range. Frequencies and percentages were used to assess types of violence, which were compared with sociodemographic characteristics using Pearson’s chi-square test or Fisher’s exact test, as appropriate. Fisher’s exact test was used when expected cell counts were less than five. The frequency of experiencing violence was determined by summing the responses corresponding to “occasionally,” “sometimes,” and “frequently.” This operational definition was used to capture any exposure to violent behaviors during clinical training, as even infrequent events may have relevant academic and emotional consequences. Given the categorical nature of the variables, no continuous scores were derived from the instrument. A logistic regression model was performed for each type of violence (non-physical, physical and sexual harassment), adjusted by age tertile, sex and year of study, which were selected a priori based on their theoretical relevance and previous literature. Odds ratios with 95% confidence intervals were obtained. Missing data were minimal (<5%) and were handled using complete case analysis. For item-level analyses (e.g., perpetrators, academic impact, and emotional consequences), the number of responses varied slightly (*n* = 71–73). No imputation procedures were performed. All analyses were performed using SPSS version 25.0 (IBM Corp., Armonk, NY, USA). A *p*-value < 0.05 was considered statistically significant.

### 2.5. Ethical Considerations

The study was approved by the Institutional Review Board of the Facultad de Ciencias de la Salud–Universidad Panamericana (registration number: CIE_PI_091_2023_1; approved on 6 November 2023), prior to the initiation of data collection. Additional approval was obtained from the Instituto Nacional de Perinatología (registration number: 2024-1-17; approved on 1 November 2024), in accordance with institutional requirements related to the study setting and data collection sites.

Informed consent was obtained electronically before participants began the online questionnaire. Each participant was assigned an ID number during data collection and analysis to guarantee confidentiality and anonymity. All data were stored in secure files with access limited to the research investigators.

Because experiencing or recalling any form of violence may cause emotional distress, participants were informed that psychological support was available if needed. The study received funding from the Fondo de Apoyo a la Investigación de la Facultad de Ciencias de la Salud 2023, Universidad Panamericana.

## 3. Results

A total of 73 nursing students participated in the study. The median age was 23 years (p25: 21, p75: 28); 38.4% aged 22 to 25 years; 86.3% were female; and most of them (43.8%) were graduate students (Table 1).

The most frequent type of violence experienced by nursing students during clinical practice was non-physical (90.4%), followed by sexual harassment (49.3%), and physical violence (17.8%). The place where nursing students had experienced violence the most is in hospitals (57.7%, *n* = 41) rather than in clinics (18.6%, *n* = 13).

Regarding non-physical violence, the most common types were non-verbal acts (e.g., “raised eyebrows”, “rolling eyes”), “being ignored or neglected”, followed by “not being treated as part of the multidisciplinary team”, although other forms of non-physical violence were also reported at lower frequencies. Figure 1 shows the distribution of non-physical violence types experienced by nursing students.

For physical violence, the most frequently reported incidents were “being pushed or shoved”, “being hit with something”, and “having something of mine deliberately damaged”, while other types of physical violence were also reported less frequently (Figure 2).

Sexual harassment included behaviors such as “sexist remarks directed at me”, “suggestive sexual gestures directed at me”, and “requests for intimate physical contact” with additional behaviors reported at lower frequencies (Figure 3).

As shown in Table 2, physical violence was significantly more frequent among students aged 22 to 25 years (*p* = 0.031), while sexual harassment also showed a significant association with age (*p* = 0.047). Non-physical violence was significantly associated with the year of study (*p* = 0.010), being more prevalent among senior and graduate students. No significant differences were observed by sex for any type of violence. In the adjusted logistic regression models (Table 3), however, none of the sociodemographic characteristics remained significantly associated with experiencing any type of violence during clinical practice.

Regarding perpetrators, nursing students reported experiencing violence during clinical practice mainly by nurses (62%), doctors (46.5%), and patients (36.6%), while other perpetrators were also reported at lower frequencies (Figure 4a).

As shown in Figure 4b, violence experienced during clinical practice had an impact on students’ academic performance. The most frequently reported impact was “considering leaving nursing” (41.4%), followed by “feeling scared to check orders for patient care” (32.4%), although other academic consequences were also reported less frequently.

Figure 4c shows the emotional consequences of violence experienced during clinical practice. “Feelings of inadequacy” were the most commonly reported emotional response (66.2%), followed by “humiliation or embarrassment” (62.3%) and “anxiety or fear” (60.6%).

## 4. Discussion

This study identified a high frequency of violence experienced by nursing students from a School of Nursing in Mexico City during clinical practice, with non-physical violence being the most prevalent form. Beyond its frequency, these findings are particularly relevant as exposure to violence during training may represent a significant threat to students’ mental well-being, learning experiences, and professional development. A considerable proportion of participants reported thoughts of leaving the profession and experiencing feelings of inadequacy, humiliation, and anxiety, highlighting the potential psychological impact of these experiences during a critical stage of professional formation. Importantly, although non-physical violence was the most frequently reported, all forms of violence identified in this study are relevant and should be considered when examining students’ clinical training experiences.

Consistent with previous research conducted in North America [16], Europe [17,18], Asia [19,20,21] and Australia [22], non-physical violence was the most frequently reported form of aggression in clinical practice. In these studies, verbal, nonverbal, and psychological aggression were also identified as predominant behaviors. However, the prevalence observed in the present study in Mexico (90%) was higher than the rates reported internationally (20–77%). The higher prevalence observed in our study can be interpreted within the context of organizational and hierarchical dynamics in clinical training environments, as previously described. In Mexico, clinical settings are often characterized by marked power asymmetries, where students occupy subordinate positions within institutional hierarchies, which limit their autonomy and shape their responses to adverse experiences. Within this context, exposure to violence may not only be tolerated but also progressively normalized as part of professional socialization, as previously described [5,8,9,23,24]. At the same time, differences in assessment tools across studies may also partially explain the variability in reported rates. From a mental health perspective, the high prevalence of non-physical violence is particularly relevant due to its widespread occurrence, as repeated exposure to these forms of aggression may accumulate over time and contribute to psychological distress [9,25]. Importantly, all forms of violence identified in this study—both physical and non-physical—represent harmful experiences that may negatively affect students’ well-being and professional development. These findings can also be interpreted through the lens of the hidden curriculum in clinical training environments. Within this context, beyond formal teaching, students may internalize observed behaviors, including patterns of communication, authority, and conflict management [11]. Repeated exposure to disrespectful or aggressive interactions—particularly when involving senior staff—may implicitly signal that such behaviors are tolerated or even expected within professional practice. This process may contribute to the normalization of violence and the perpetuation of hierarchical dynamics across generations of healthcare professionals [9,11].

Sociodemographic variables such as age and year of study showed statistically significant associations in the bivariate analysis with different types of violence, which may suggest that students who are younger or at earlier stages of their academic training may experience situations of violence during clinical rotations more frequently. These findings are consistent with previous studies, in which junior nursing students reported experiencing vertical violence more frequently, which is attributed to their subordinate position in clinical hierarchies and their limited experience in hospital settings [26]. Consistent with these findings, studies conducted in the Asian population show that exposure to violence occurs primarily during early clinical training, especially in students with little experience in clinical settings [21]. Nevertheless, none of the sociodemographic variables was associated with experiencing violence in multivariable models. This finding suggests that contextual and organizational factors may be more strongly influencing violence during clinical practice than individual characteristics [1].

Violence experienced by nursing students in this study in Mexico affected both academic and emotional aspects. Among the academic consequences, the intention to abandon nursing surged as the most frequently reported outcome. At the emotional level, students frequently reported feelings of inadequacy, embarrassment, and fear following experiences of violence. The first of these findings is consistent with reports from a study conducted in Australia, where exposure to violence led many nursing students to experience stress and anxiety that could cause them to consider abandoning [22]. Similarly, studies conducted in Asian populations identified emotions such as fear, a decrease in self-confidence, and feelings of inadequacy after experiencing violence during clinical rotations [21]. These findings highlight challenges described by the World Health Organization regarding the retention of nursing professionals and their effects on global healthcare [14].

The identification of nurses and physicians as primary perpetrators in our study is noteworthy, as several international studies have reported patients as the main source of violence toward nursing students [4,18,27]. In many of these contexts, patient-related aggression, particularly verbal hostility, has been described as the most frequent form of violence during clinical training. Although patients were also identified as perpetrators in the present study, the prominent role of healthcare professionals suggests that violence may not be limited to patient-related interactions but may also emerge within professional hierarchies [28]. This finding may reflect the influence of organizational culture, power asymmetries, and role expectations within clinical environments, where students typically occupy subordinate positions. In such contexts, exposure to violence may be shaped by vertical dynamics that reinforce authority gradients and limit students’ autonomy. These patterns may contribute to the normalization of violence within clinical training environments and influence professional socialization processes, potentially perpetuating these behaviors across generations of healthcare professionals [29,30].

Evidence regarding violence experienced by nursing students in Latin American contexts remains limited [4,31], as most available research originates from high-income countries in North America, Europe, and Asia. This scarcity of regional data limits the understanding of how sociocultural and organizational factors specific to Latin American healthcare systems may shape students’ experiences during clinical practice. In Mexico, the scope of nursing practice and levels of professional autonomy differ from those reported in countries such as the United States or the United Kingdom, where nurses often hold greater decision-making authority and institutional recognition. These structural differences may influence interpersonal dynamics within clinical settings and potentially affect how violence is manifested, perceived, and reported during training [8,29,32]. Taken together, these findings are relevant to an international readership because they suggest that violence toward nursing students is not only an individual or isolated problem, but also a context-dependent feature of clinical training environments shaped by organizational hierarchy, professional autonomy, and reporting culture. Furthermore, although participants in this study were enrolled in a private nursing school and completed clinical placements across both public and private healthcare institutions, reports of violence were observed across settings. This finding suggests that exposure to violence may not be confined to a particular institutional sector but could reflect broader systemic and cultural patterns within clinical training environments.

From an educational perspective, our findings suggest the need to strengthen support systems within clinical training environments. Considering the high frequency of non-physical violence and its academic and emotional impact, nursing programs should ensure that students have access to clear reporting mechanisms and consistent mentorship during clinical practice. Providing structured opportunities to discuss professional boundaries, hierarchical dynamics, and respectful communication may also help students better navigate complex interpersonal situations. In light of these findings, educational institutions and healthcare facilities should work jointly to promote respectful interactions, address hierarchical dynamics, and model professional conduct within clinical learning environments. Addressing violence during training is particularly important, as early negative experiences may influence students’ confidence, sense of belonging, and even their intention to remain in the profession [1,6].

Given the cross-sectional design and small sample size, these findings should be interpreted cautiously. The relatively small sample size may have limited the statistical power to detect significant associations, particularly in multivariable analyses, increasing the possibility of type II error. Additionally, the limited number of events for some outcomes may have affected the stability of the regression models, increasing the risk of overfitting and contributing to the wide confidence intervals observed. This is reflected in the large odds ratios and wide confidence intervals observed in Table 3. Therefore, the results of multivariable analyses should be interpreted with caution and considered exploratory. Furthermore, although the presence of researchers during data collection was limited to providing clarification and technical support, the group-based setting may have introduced a degree of social desirability bias, potentially influencing participants’ responses despite the measures taken to ensure anonymity. Additionally, the definition of violence as any occurrence other than “never” may have influenced the prevalence estimates and should be considered when comparing results across studies using different thresholds. The use of convenience sampling may limit the generalizability of the results. However, participants completed clinical placements in both public and private healthcare institutions, which may increase the relevance of the findings to similar educational contexts in Mexico. The study does not allow for causal inferences; however, the consistency of reports across academic levels and clinical contexts highlights the need for further research examining organizational culture, hierarchical structures, and professional socialization processes within Mexican healthcare institutions. Expanding research in Latin American settings will be essential to inform context-specific educational and institutional strategies aimed at promoting safer clinical learning environments.

## 5. Conclusions

This study identified a high frequency of violence experienced by nursing students from a School of Nursing in Mexico City during clinical practice, with non-physical violence being the most frequently reported form. Exposure to violence was related to academic and emotional consequences, highlighting the need to strengthen reporting mechanisms and promote safer clinical learning environments to support students’ well-being and professional development.

These findings have important implications for nursing students’ mental health and well-being. Repeated exposure to violence during clinical training may contribute to emotional distress, reduced self-confidence, and intentions to leave the profession, underscoring the importance of early interventions. Addressing violence requires not only strengthening reporting mechanisms but also critically examining hierarchical structures and organizational cultures that may enable or normalize such experiences. Promoting safe and supportive learning environments should be considered a shared responsibility between educational institutions and healthcare settings.

Future research should further explore how exposure to violence during clinical training affects nursing students’ mental health over time, as well as the effectiveness of interventions aimed at reducing its impact. Longitudinal and multi-institutional studies would be valuable to better understand the long-term consequences of these experiences and to inform strategies that promote psychological well-being and retention in the nursing workforce.

## Figures and Tables

**Table 1:** General characteristics of nursing students (*n* = 73).

	*n* (%)
Age	
≤21 years	21 (28.8)
22 to 25 years	28 (38.4)
≥26 years	24 (32.9)
Sex	
Woman	63 (86.3)
Man	10 (13.7)
Year of study	
1st year	7 (9.6)
2nd year	10 (13.7)
3rd year	3 (4.1)
4th year	13 (17.8)
Intern	8 (11.0)
Graduate student	32 (43.8)

**Table 2:** Types of violence experienced by nursing students according to sociodemographic characteristics (*n* = 73).

	Non-Physical Violence		Physical Violence		Sexual Harassment	
	Yes *n* (%)	*p*-Value	Yes *n* (%)	*p*-Value	Yes *n* (%)	*p*-Value
Age		0.342		0.031		0.047
≤21 years	20 (30.3)		0 (0)		6 (16.7)	
22 to 25 years	26 (39.4)		8 (61.5)		18 (50.0)	
≥26 years	20 (30.3)		5 (38.5)		12 (33.3)	
Sex		0.229		0.845		0.526
Female	58 (87.9)		11 (84.6)		32 (88.9)	
Male	8 (12.1)		2 (15.4)		4 (11.1)	
Year of study		0.010		0.216		0.061
1st year	6 (9.1)		0 (0)		0 (0)	
2nd year	10 (15.2)		0 (0)		4 (11.1)	
3rd year	1 (1.5)		0 (0)		1 (2.8)	
4th year	13 (19.7)		3 (23.1)		8 (22.2)	
Intern	8 (12.1)		3 (23.1)		6 (16.7)	
Graduate student	28 (42.4)		7 (53.8)		17 (47.2)	

Percentages are presented by column. Participants were considered to have experienced violence if their responses were “occasionally”, “sometimes”, or “often”. Comparisons between those who experienced and those who did not experience each type of violence were performed using Pearson’s chi-square test or Fisher’s exact test when expected cell counts were <5.

**Table 3:** Logistic regression models for types of violence experienced by nursing students according to sociodemographic characteristics (*n* = 73).

	Non-Physical Violence			Physical Violence			Sexual Harassment		
Variable	OR	95% CI	*p*-Value	OR	95% CI	*p*-Value	OR	95% CI	*p*-Value
Age									
≤21 years	11.1	0.3–389	0.185	0	0	0.998	0.4	0.1–2.8	0.281
22 to 25 years	5.3	0.3–93	0.252	1.3	0.2–7.0	0.774	1.6	0.4–7.1	0.516
≥26 years	Ref.	Ref.	Ref.	Ref.	Ref.	Ref.	Ref.	Ref.	Ref.
Sex									
Female	2.9	0.4–19.3	0.285	0.9	0.1–5.3	0.877	1.7	0.4–6.9	0.496
Male	Ref.	Ref.	Ref.	Ref.	Ref.	Ref.	Ref.	Ref.	Ref.
Year of study									
Undergraduate student	0.3	0.02–5.8	0.423	1.3	0.2–7.4	0.742	1.1	0.2–5.1	0.916
Graduate student	Ref.	Ref.	Ref.	Ref.	Ref.	Ref.	Ref.	Ref.	Ref.

OR: odds ratio; 95% CI: confidence interval; Ref.: category of reference.

## Data Availability

The data presented in this study are available from the corresponding authors upon reasonable request due to privacy and ethical restrictions.

## Public Involvement Statement

No public involvement in any aspect of this research.

## Guidelines and Standards Statement

This manuscript was drafted against the Strengthening the Reporting of Observational Studies in Epidemiology (STROBE) Statement for cross-sectional studies.
